# Icatibant, another piece of the therapeutic puzzle regarding hemodynamic side effects of angiotensin-converting enzyme inhibitors

**DOI:** 10.1186/s13054-019-2571-x

**Published:** 2019-08-28

**Authors:** Patrick M. Honore, David De Bels, Leonel Barreto Gutierrez, Sebastien Redant, Andrea Gallerani, Willem Boer

**Affiliations:** 1 ICU Department, Centre Hospitalier Universitaire Brugmann, 1020 Brussels, Belgium; 2 Department of Anesthesiology, Intensive Care Medicine, Emergency Medicine & Pain Medicine, Ziekenhuis Oost- Limburg, Genk, Belgium

With interest we read the recent paper by Charbonneau et al. tackling side effects of angiotensin-converting enzyme inhibitor (ACEI) therapy during hypovolemic shock in mice by using icatibant, a specific bradykinin beta 2 receptor antagonist [1]. They demonstrated not only the relative inefficacy of vasopressors in this setting, but also the impressive results utilizing icatibant in a hypovolemic mice model [2]. We recently reported upon the efficacy of rescue naloxone (2 mg) followed by a 24-h infusion (0.04 mg/kg/h) in a mechanically ventilated patient, subject to ACEI intoxication with severe hemodynamic instability and severe bradycardia. To the best of our knowledge, this was the first case reporting an impressive effect of naloxone therapy, as in most cases effects are limited or absent [3]. Modification of baroreflexes, parasympathetic activation, or discontinuation of angiotensin II-mediated vagal inhibition have been proposed as potential mechanisms to explain the lack of compensatory tachycardia following ACEI-induced blood pressure fall [3]. In vitro, ACEI inhibits enkephalinase and thus increases endogenous opioid levels, themselves reducing baroreflex sensitivity [4]. One study has demonstrated a higher baseline heart rate in healthy volunteers treated with naloxone plus captopril in comparison to the group receiving captopril alone [4]. This suggests that using opioid antagonists such as naloxone is an interesting therapeutic option in case of ACEI intoxication [5]. With pharmaco-economic considerations in mind, a two-tier therapeutic regimen could be applied in case of hemodynamic and rhythmic complications due to ACEI: first, using a naloxone bolus followed by a 24-h infusion. In case of lack of response to naloxone, which is often the case, a second-line drug such as icatibant could be applied as rescue therapy.

## Data Availability

Not applicable.

## Abbreviation

ACEI: Angiotensin-converting enzyme inhibitor
